# Supplementary material for: Mapping the Energetic Costs of Free-Swimming Gilthead Sea Bream (Sparus aurata), a Key Species in European Marine Aquaculture
Source: Biology (Basel). 2021 Dec 20;10(12):1357. doi: 10.3390/biology10121357 (PMC8698635; doi:10.3390/biology10121357)
Supplement: Supplementary file 1 [file biology-10-01357-s001.zip › biology-1484383-supplementary.pdf]

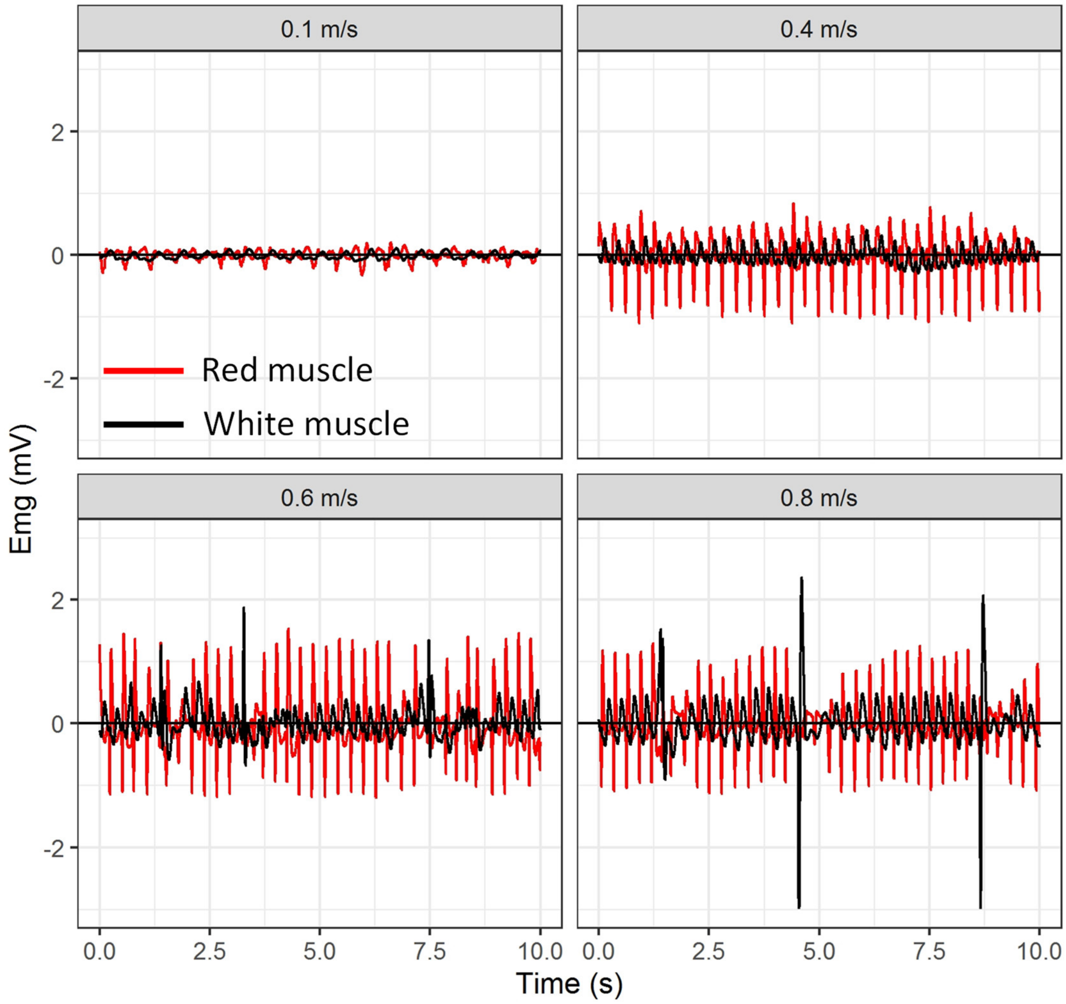

Figure S1. Sample of 10 seconds EMG signal (Millivolts, mV) of both red (red line) and white muscles (black line) at four different water speed: 0.1, 0.4, 0.6 and 0.8 m/s recorded during a critical swimming test in Gilt-head Sea bream *(Sparus aurata)*.

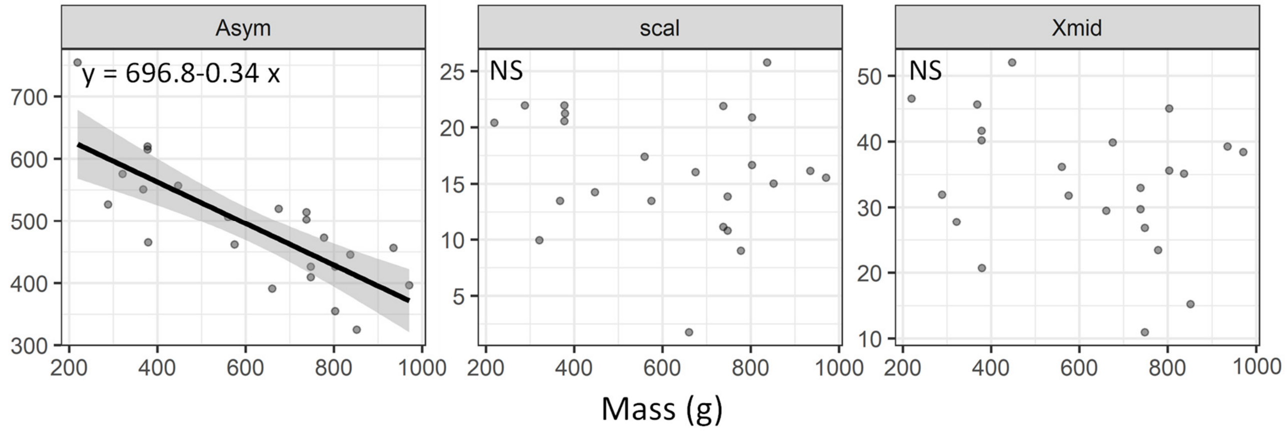

Figure S2. Linear regressions of the parameters (Asym, xmid and scal) from the SSlogis model obtained for each fish as function of mass in Gilt-head Sea bream (*Sparus aurata*; n=26). The black line represents a significant linear regression between parameter and fish mass, and the grey shape represents the 95 % confidence interval. Not significant (NS) is mentioned for each non significative parameter.

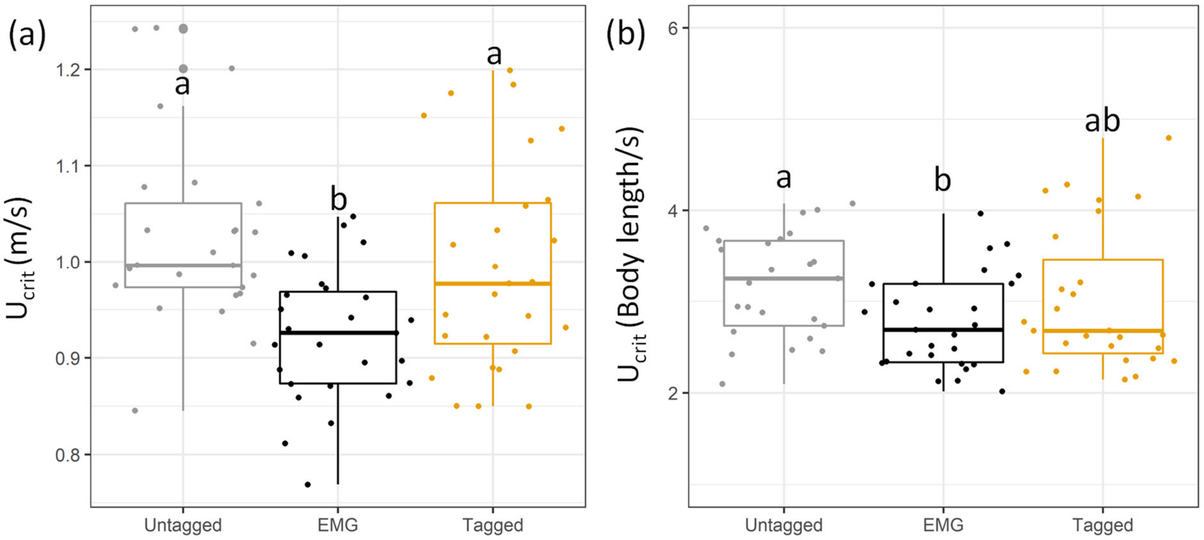

Figure S3. Swimming performances during U_{crit} trial according to their condition in Gilt-head Sea bream (*Sparus aurata*; Untagged, n=25, grey dots; EMG, n=27, black dots or tagged, n=27, orange dots). **(a)** Absolute and **(b)** relative U_{crit} values (m/s and BL/s) as function (untagged fish, n=25, grey points; tagged fish, n=27, orange points). The central line of the boxplot indicates the median and the boxes the quartiles, with the whiskers covering 95% of the values. Different letters indicate significant statistical difference between conditions (Kruskal-Wallis followed by followed by Dunn's-test adjusted by Bonferroni, $p < 0.05$).

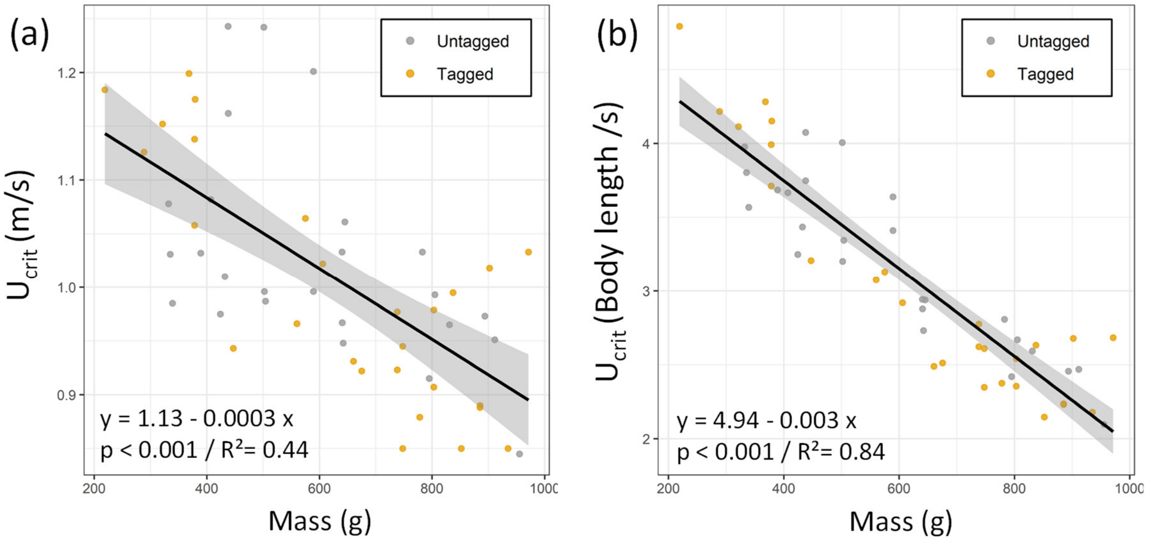

Figure S4. Swimming performances of Gilt-head Sea bream (*Sparus aurata*) during U_{crit} trial. (**a**) Absolute and (**b**) relative U_{crit} values (m/s and BL/s) as function of fish mass (g) and tagging condition (grey dots, n=25 grey dots; orange dots, n=27, tagged fish). The black line represents the significant linear regression between the U_{crit} and mass, and grey shape indicates the 95 % confidence interval of the regression. Equation, p values and R^2 are reported for each variable and the interaction of both (Linear regression, $p < 0.05$).

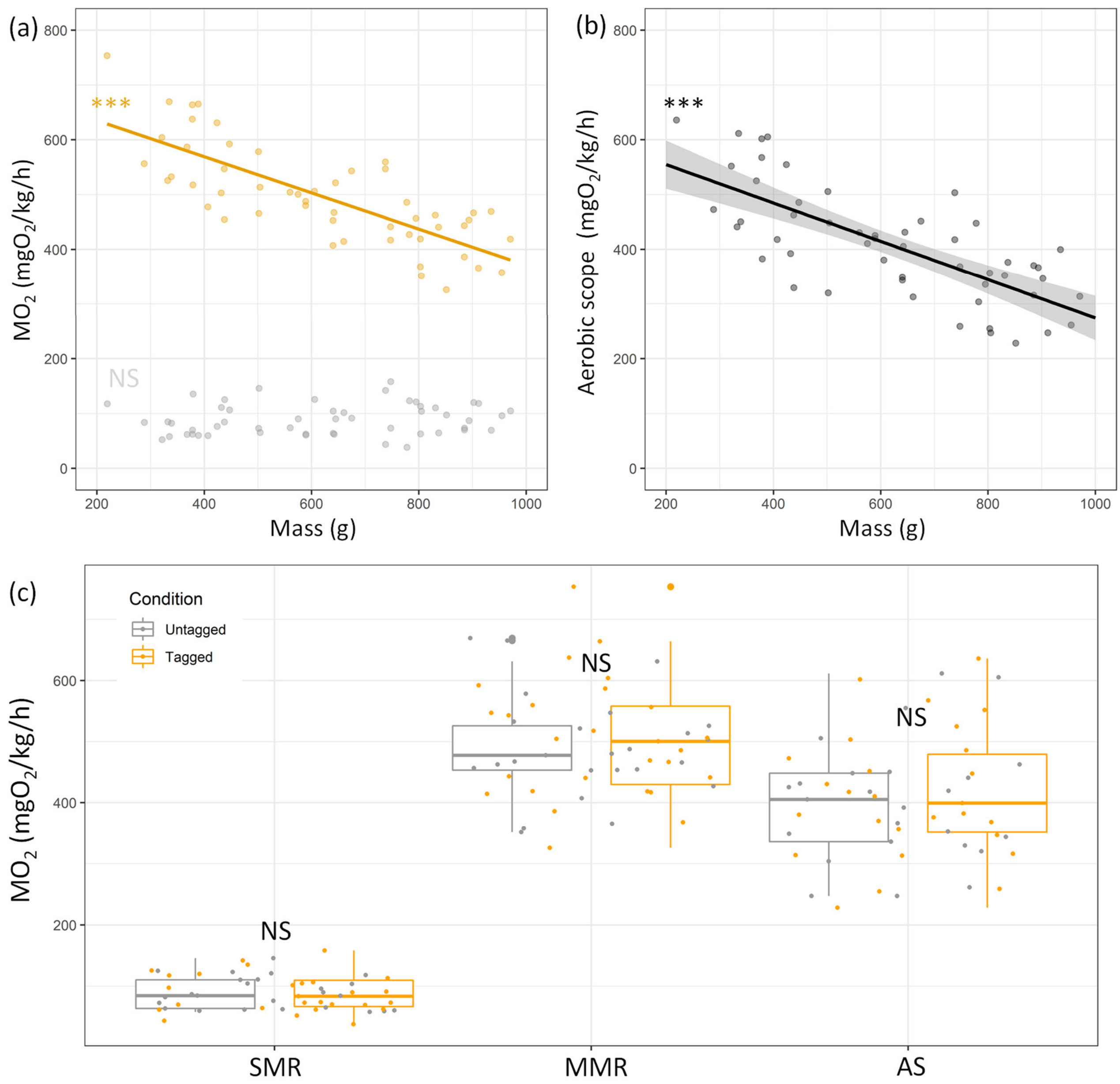

Figure S5. Metabolic variables as a function of fish mass in Gilt-head Sea bream and tagging condition (*Sparus aurata***;** n = 52). **(a)** Standard and maximum metabolic rates (SMR, grey dots and MMR, orange dots); **(b)** Aerobic scope (AS; MO_2, mgO_2/kg/h) and **(c)** metabolic traits (SMR, MMR and AS) depending on tagging condition (untagged in dark grey and tagged in orange). Each dot represents the metabolic variables of a single fish. For panel (a), the grey line represents the SMR and the orange line represents linear regression for the MMR values. For panel (b), the black line represents linear regression for the AS and the grey shape represents the 95 % confidence interval. For panels **(a)** and **(b)**, significance of the linear regressions is indicated according to Linear regression, and for panel **(c)** according to Student t-test (***: p<0.001 / not significant (NS)).

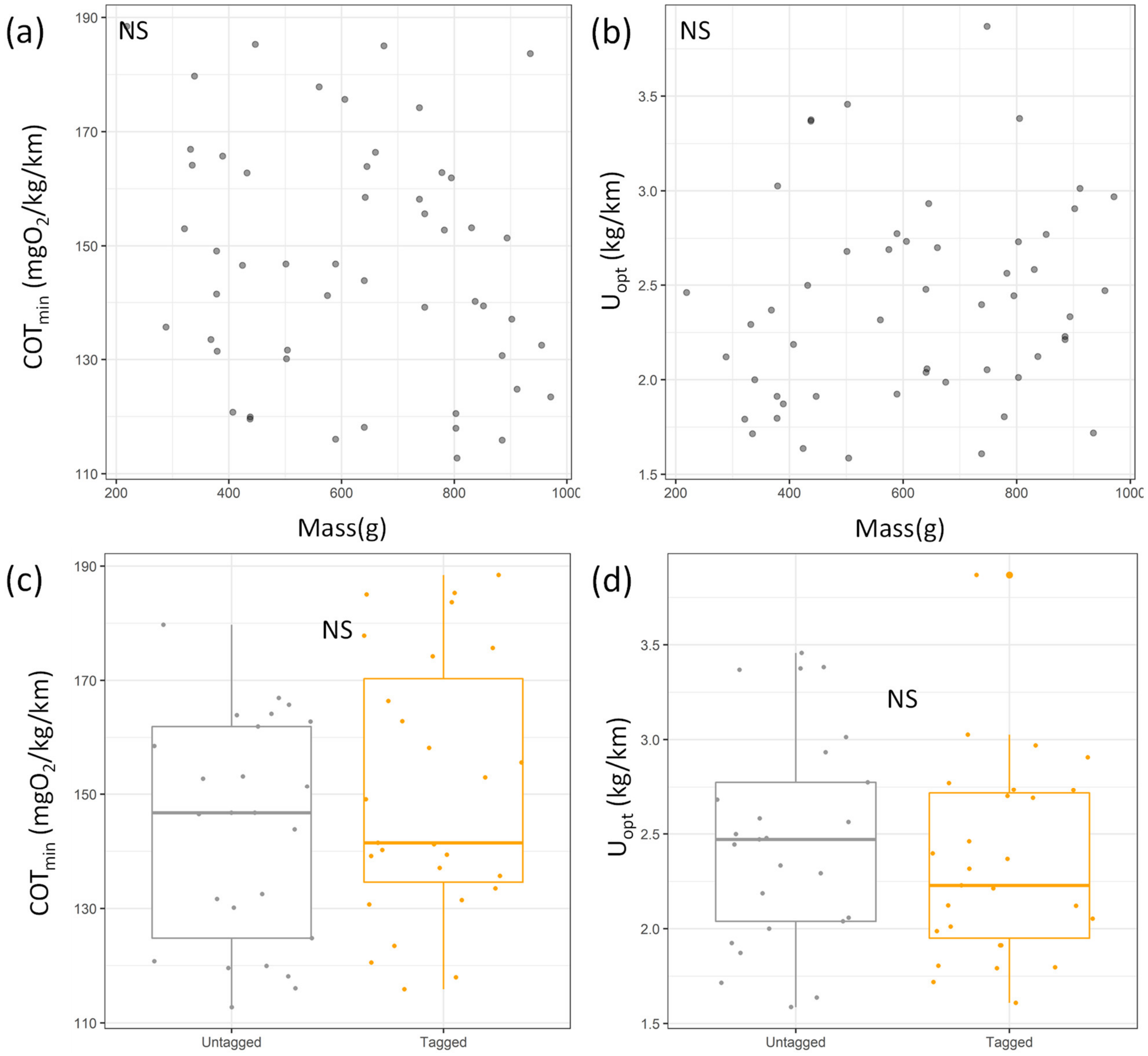

Figure S6. Swimming efficiency and cost of transport in Gilt-head Sea bream (*Sparus aurata*; n=52 fish). **(a)** COT_{min} (mgO_2/kg/km) and **(b)** U_{opt} (kg/km) as function of fish mass; and **(c)** COT_{min} (mgO_2/kg/km) and **(d)** U_{opt} (kg/km) as function of fish tagging procedure (untagged fish, n=25, grey points; tagged fish, n=27, orange points). The central line of the boxplot indicates the median and the boxes the quartiles, with the whiskers covering 95% of the values. For panels **(a)** and **(b)**, statistics are indicated according to linear regressions, and for panels **(c)** and **(d)**, statistics are indicated according to Student t-test (Not significant (NS): $p > 0.05$).

Table S1. Outputs of linear model for fitting the swimming performances (absolute and relative Ucrit) as a function of mass (g) and condition (untagged or tagged) in Gilt-head Sea bream (Sparus aurata). Associated R^2 are 0.44 and 0.83 for absolute and relative Ucrit respectively.

**Absolute Ucrit**				
**Parameter**	**Estimate**	**Std. error**	**t value**	**p value**
(intercept)	1.24	0.047	26.66	<0.001
Mass	-0.0004	0.000068	-5.58	<0.001
Condition	-0.066	0.069	-0.96	0.34
Mass : condition	0.0013	0.00011	1.24	0.22
**Relative Ucrit**				
**Parameter**	**Estimate**	**Std. error**	**t value**	**p value**
(intercept)	5.07	0.16	30.96	<0.001
Mass	-0.003	0.002	-13.36	<0.001
Condition	-0.33	0.24	-1.35	0.18
Mass : condition	0.0006	0.004	1.53	0.13

Table S2. Outputs of sigmoid model for fitting the MO2 as a function of swimming activity (AU) during Ucrit for each tagged Gilt-head Sea bream (Sparus aurata; n = 23 fish). P value and R^2 are shown for each model. Estimate, standard error and level of significance are presented for each parameter of the model (Asym, xmid and scal) are indicated. For the level of significance, not significant (NS) is indicated for p>0.1, # for 0.05 <p<0.1, * for p<0.05, ** for p<0.01 and *** for p<0.001.

		Estimate ± std. error (significance)				
**Fish ID**	**Mass**	**Asym**	**Xmid**	**scal**	**p value**	**R^2**
1	219	754.45 ± 38.86 (***)	46.56 ± 3.39 (***)	20.41 ± 3.1 (***)	<0.001	0.93
2	288.4	526.54 ± 18.12 (***)	31.94 ± 2.57 (***)	21.94 ± 3.08 (***)	<0.001	0.98
3	368	551.08 ± 11.88 (***)	45.63 ± 1.32 (***)	13.49 ± 1.48 (***)	<0.001	0.98
4	379	465.05 ± 12.20 (***)	20.7 ± 2.20 (***)	21.24 ± 3.68 (**)	<0.001	0.99
5	321.2	575.67 ± 12.92 (***)	27.76 ± 1.64 (***)	9.94 ± 1.75 (**)	<0.001	0.96
6	378	614.98 ± 30.09 (***)	40.22 ± 4.15 (***)	21.94 ± 4.10 (**)	<0.001	0.95
7	378	619.6 ± 12.82 (***)	41.67 ± 1.61 (***)	20.55 ± 1.46 (***)	<0.001	0.99
8	447	556.59 ± 46.68 (***)	52.04 ± 3.80 (***)	14.26 ± 3.82 (*)	<0.001	0.93
9	575	461.88 ± 26.43 (***)	31.79 ± 2.84 (***)	13.49 ± 3.42 (*)	<0.001	0.95
10	560	506.5 ± 32.83 (***)	36.18 ± 5.42 (**)	17.4 ± 5.64 (*)	<0.001	0.9
11	675	519.85 ± 28.90 (***)	39.85 ± 3.20 (***)	16.02 ± 3.63 (*)	<0.001	0.96
12	660.2	390.78 ± 20.41 (***)	29.47 ± 0.58 (***)	1.77 ± 0.84 (NS)	<0.001	0.95
14	777.87	472.44 ± 23.21 (***)	23.45 ± 2.20 (***)	9.02 ± 2.59 (*)	<0.001	0.95
15	738	514.34 ± 25.41 (***)	29.74 ± 3.27 (***)	21.9 ± 4.52 (**)	<0.001	0.98
16	738	501.86 ± 11.31 (***)	32.99 ± 1.18 (***)	11.13 ± 1.21 (***)	<0.001	0.99
17	747.59	426.07 ± 17.44 (***)	26.81 ± 1.96 (***)	10.80 ± 2.72 (*)	<0.001	0.98
18	747.59	408.95 ± 5.00 (***)	10.94 ± 1.31 (**)	13.87 ± 1.56 (**)	<0.001	0.99

19	851.7	324.88 ± 25.99 (**)	15.23 ± 4.52 (*)	15 ± 5.90 (#)	<0.001	0.98
22	837	445.15 ± 6.64 (***)	35.13 ± 12.01 (*)	25.76 ± 17.16 (NS)	<0.001	0.78
23	803	354.94 ± 36.39 (***)	35.59 ± 5.91 (**)	20.89 ± 8.36 (#)	<0.001	0.97
24	803	426.03 ± 9.19 (***)	45.06 ± 1.31 (***)	16.66 ± 1.43 (***)	<0.001	0.99
25	935	456.41 ± 5.82 (***)	39.28 ± 0.70 (***)	16.14 ± 0.87 (***)	<0.001	0.99
26	971	396.22 ± 11.58 (***)	38.4 ± 1.67 (***)	15.53 ± 2.5 (**)	<0.001	0.99

Table S3. Outputs of sigmoid model for fitting the MO2 as a function of swimming speed (m/s) during Ucrit for each Gilt-head Sea bream (Sparus aurata; n=25 untagged and n=27 tagged fish). P value and R^2 are shown for each model. (a) Estimate, standard error and level of significance are presented for each parameter of the model (Asym, xmid and scal) are also indicated. For the level of significance, not significant (NS) is indicated for p>0.1, # for 0.05 <p<0.1, * for p<0.05, ** for p<0.01 and *** for p<0.001.

			Estimate ± std. error (significance)				
**Fish ID**	**Condition**	**Mass (g)**	**A_{sym}**	**x_{mid}**	**scal**	**p value**	**R^2**
1	Untagged	332	531.61 ± 13.78 (***)	0.29 ± 0.02 (***)	0.17 ± 0.02 (***)	<0.001	0.99
2	Untagged	335	731.33 ± 68.43 (***)	0.52 ± 0.05 (***)	0.21 ± 0.03 (**)	<0.001	0.97
3	Untagged	339	546.31 ± 56.21 (***)	0.31 ± 0.06 (**)	0.18 ± 0.06 (*)	<0.001	0.92
4	Untagged	389	750.47 ± 95.84 (**)	0.56 ± 0.07 (**)	0.23 ± 0.05 (**)	<0.001	0.97
5	Untagged	437.8	658.06 ± 191.5 (*)	0.68 ± 0.24 (*)	0.36 ± 0.13 (*)	<0.001	0.91
6	Untagged	437.6	544.67 ± 104.19 (**)	0.5 ± 0.18 (*)	0.41 ± 0.13 (*)	<0.001	0.97
7	Untagged	424	840.94 ± 329.03 (#)	0.66 ± 0.26 (#)	0.29 ± 0.12 (#)	<0.001	0.89
8	Untagged	432	538.16 ± 52.24 (***)	0.34 ± 0.06 (**)	0.25 ± 0.06 (**)	<0.001	0.97
9	Untagged	407	525.81 ± 65.88 (***)	0.51 ± 0.08 (***)	0.25 ± 0.06 (**)	<0.001	0.95
10	Untagged	501.2	597.05 ± 44.32 (***)	0.45 ± 0.05 (***)	0.23 ± 0.05 (**)	<0.001	0.94

11	Untagged	589.3	515.76 ± 65.35 (***)	0.52 ± 0.09 (***)	0.26 ± 0.07 (**)	<0.001	0.93
12	Untagged	589.3	508.63 ± 39.1 (***)	0.38 ± 0.04 (***)	0.19 ± 0.04 (**)	<0.001	0.97
13	Untagged	502	882.32 ± 1066.7 (NS)	0.93 ± 1.36 (NS)	0.58 ± 0.41 (NS)	<0.001	0.95
14	Untagged	504	610.34 ± 283.15 (#)	0.55 ± 0.28 (NS)	0.26 ± 0.14 (NS)	<0.001	0.91
15	Untagged	640.4	460.91 ± 61.27 (***)	0.46 ± 0.09 (**)	0.25 ± 0.06 (**)	<0.001	0.98
16	Untagged	640.21	489.1 ± 37.95 (***)	0.35 ± 0.05 (**)	0.27 ± 0.05 (**)	<0.001	0.99
17	Untagged	644.89	541.14 ± 70.86 (**)	0.35 ± 0.07 (**)	0.22 ± 0.1 (#)	<0.001	0.94
18	Untagged	642	483.9 ± 63.74 (**)	0.34 ± 0.07 (**)	0.18 ± 0.06 (*)	<0.001	0.94
19	Untagged	794.86	508.47 ± 84.42 (**)	0.32 ± 0.11 (*)	0.27 ± 0.1 (*)	<0.001	0.97
20	Untagged	782.5	438.94 ± 42.73 (***)	0.21 ± 0.06 (*)	0.23 ± 0.08 (*)	<0.001	0.96
21	Untagged	830.77	520.36 ± 130.67 (*)	0.37 ± 0.17 (#)	0.28 ± 0.13 (#)	<0.001	0.96
22	Untagged	805	375.87 ± 50.49 (**)	0.26 ± 0.09 (*)	0.27 ± 0.11 (#)	<0.001	0.97
23	Untagged	893.6	484.94 ± 99.33 (**)	0.35 ± 0.12 (*)	0.23 ± 0.1 (#)	<0.001	0.94
24	Untagged	911.3	415.58 ± 116.31 (*)	0.3 ± 0.2 (NS)	0.33 ± 0.18 (NS)	<0.001	0.97
25	Untagged	955	418.26 ± 91.19 (*)	0.34 ± 0.14 (#)	0.28 ± 0.1 (#)	<0.001	0.99
1	Tagged	219	816.95 ± 81.93 (***)	0.5 ± 0.07 (***)	0.28 ± 0.058 (**)	<0.001	0.92
2	Tagged	288.4	643.67 ± 98.66 (***)	0.57 ± 0.11 (**)	0.3 ± 0.07 (**)	<0.001	0.96
3	Tagged	368	630.49 ± 60.21 (***)	0.55 ± 0.07 (***)	0.25 ± 0.05 (**)	<0.001	0.94
4	Tagged	379	746.1 ± 231.26 (*)	0.76 ± 0.33 (#)	0.51 ± 0.14 (*)	<0.001	0.99
5	Tagged	321.2	611.8 ± 13.4 (***)	0.41 ± 0.01 (***)	0.17 ± 0.01 (***)	<0.001	0.99
6	Tagged	378	727.06 ± 91.1 (***)	0.56 ± 0.08 (***)	0.25 ± 0.05 (**)	<0.001	0.94
7	Tagged	378	732.34 ± 62.68 (***)	0.58 ± 0.06 (***)	0.24 ± 0.03 (***)	<0.001	0.97

8	Tagged	447	691.44 ± 151.74 (*)	0.46 ± 0.14 (*)	0.27 ± 0.08 (*)	<0.001	0.96
9	Tagged	575	553.97 ± 125.34 (**)	0.46 ± 0.16 (*)	0.28 ± 0.11 (#)	<0.001	0.93
10	Tagged	560	508.17 ± 13.99 (***)	0.25 ± 0.02 (***)	0.14 ± 0.019 (***)	<0.001	0.99
11	Tagged	675	579.9 ± 74.2 (**)	0.35 ± 0.07 (**)	0.21 ± 0.06 (*)	<0.001	0.96
12	Tagged	660.2	418.22 ± 34.37 (***)	0.18 ± 0.04(*)	0.16 ± 0.06 (#)	<0.001	0.96
13	Tagged	605.86	519.37 ± 38.72 (***)	0.24 ± 0.04 (**)	0.21 ± 0.06 (*)	<0.001	0.96
14	Tagged	777.87	495.23 ± 55.15 (***)	0.34 ± 0.06 (**)	0.14 ± 0.05 (#)	<0.001	0.89
15	Tagged	738	910.7 ± 533.14 (NS)	0.72 ± 0.51 (NS)	0.43 ± 0.17 (#)	<0.001	0.97
16	Tagged	738	564.92 ± 46.99 (***)	0.41 ± 0.04 (***)	0.17 ± 0.03 (**)	<0.001	0.97
17	Tagged	747.59	452.71 ± 54.16 (***)	0.29 ± 0.07 (*)	0.18 ± 0.07 (#)	<0.001	0.96
18	Tagged	747.59	492.12 ± 167.85 (#)	0.26 ± 0.22 (NS)	0.35 ± 0.28 (NS)	<0.001	0.98
19	Tagged	851.7	331.18 ± 21.55 (***)	0.15 ± 0.03(*)	0.17 ± 0.05 (*)	<0.001	1
20	Tagged	885	640.84 ± 398.29 (NS)	0.6 ± 0.42 (NS)	0.33 ± 0.17 (NS)	<0.001	0.94
21	Tagged	885	556.85 ± 292.46 (NS)	0.62 ± 0.38 (NS)	0.33 ± 0.15 (NS)	<0.001	0.98
22	Tagged	837	450.01 ± 29.21 (***)	0.32 ± 0.04 (***)	0.18 ± 0.04 (**)	<0.001	0.97
23	Tagged	803	574.55 ± 321.2 (NS)	0.64 ± 0.53 (NS)	0.46 ± 0.21 (#)	<0.001	0.99
24	Tagged	803	538.33 ± 86.83 (**)	0.56 ± 0.11 (**)	0.28 ± 0.06 (**)	<0.001	0.98
25	Tagged	935	481.78 ± 15.77 (***)	0.28 ± 0.02 (***)	0.16 ± 0.02 (**)	<0.001	1
26	Tagged	971	490.05 ± 106.94 (**)	0.44 ± 0.17 (#)	0.34 ± 0.13 (*)	<0.001	0.97
27	Tagged	902	579.85 ± 137.19 (**)	0.49 ± 0.2 (#)	0.37 ± 0.13 (*)	<0.001	0.97

Table S3. (**b**) Outputs of sigmoid model for fitting the MO2 as a function of swimming speed (m/s) during Ucrit for each Gilt-head Sea bream (Sparus aurata; n=25 untagged and n=27 tagged fish). P value and R^2 are shown for each model. (a) Estimate, standard error and level of significance are presented for each parameter of the model (Asym, xmid and scal) are also indicated. For the level of significance, not significant (NS) is indicated for p>0.1, # for 0.05 <p<0.1, * for p<0.05, ** for p<0.01 and *** for p<0.001.

Fish ID	Condition	Mass (g)	SMR	MMR	AS
1	Untagged	332	84.96	525.86	440.9
2	Untagged	335	58.1	669.51	611.41
3	Untagged	339	82.4	532.85	450.45
4	Untagged	389	60.11	665.36	605.25
5	Untagged	437.8	84.36	547.12	462.76
6	Untagged	437.6	125.25	454.64	329.4
7	Untagged	424	76.37	631.18	554.81
8	Untagged	432	111.13	503.23	392.1
9	Untagged	407	59.59	477.63	418.04
10	Untagged	501.2	73.04	578.4	505.36
11	Untagged	589.3	60.6	480.21	419.6
12	Untagged	589.3	62.46	487.86	425.4
13	Untagged	502	145.75	465.69	319.94
14	Untagged	504	65.29	513.56	448.26
15	Untagged	640.4	63.83	407.24	343.41
16	Untagged	640.21	104.49	452.94	348.46
17	Untagged	644.89	90.19	521.47	431.29
18	Untagged	642	62.12	467.51	405.39
19	Untagged	794.86	121.11	456.7	335.59

20	Untagged	782.5	123.29	427.03	303.73
21	Untagged	830.77	110.41	462.54	352.13
22	Untagged	805	104.02	351.25	247.23
23	Untagged	893.6	87.09	453.44	366.35
24	Untagged	911.3	118.31	365.48	247.17
25	Untagged	955	96.1	357.41	261.3
1	Tagged	219	117.69	753.71	636.02
2	Tagged	288.4	83.82	556.6	472.78
3	Tagged	368	61.89	586.81	524.92
4	Tagged	379	135.26	517.62	382.37
5	Tagged	321.2	52.36	604.14	551.78
6	Tagged	378	69.98	637.57	567.59
7	Tagged	378	62.02	663.95	601.93
8	Tagged	447	106.6	592.3	485.7
9	Tagged	575	90.04	500.58	410.54
10	Tagged	560	74.1	504.62	430.52
11	Tagged	675	91.55	543.17	451.62
12	Tagged	660.2	101.72	414.52	312.8
13	Tagged	605.86	125.67	506.1	380.43
14	Tagged	777.87	38.38	485.94	447.57
15	Tagged	738	142.09	559.62	417.53
16	Tagged	738	43.74	547.19	503.45

17	Tagged	747.59	73.32	441.48	368.16
18	Tagged	747.59	158.2	417.03	258.83
19	Tagged	851.7	97.53	325.74	228.21
20	Tagged	885	73.27	443.28	370.01
21	Tagged	885	70.34	386.18	315.84
22	Tagged	837	64.46	440.66	376.15
23	Tagged	803	113.25	367.94	254.69
24	Tagged	803	62.8	418.87	356.06
25	Tagged	935	69.64	469.22	399.59
26	Tagged	971	104.75	418.51	313.76
27	Tagged	902	120.25	466.86	346.61

Table S4. Outputs of model for fitting the cost of transport (COT, mgO2/kg/lm) as function of speed (km/h) during the Ucrit trial for each Gilt-head Sea bream (Sparus aurata; n = 52). P value and R^2 were shown for each model, and estimate, standard error and level of significance of each parameter of each model (a and b) are presented, as well the estimated values of Uopt and and COTmin.

			Estimate ± std. error (significance)					
**Fish ID**	**Condition**	**Mass (g)**	**a**	**b**	**P value**	**R^2**	**U_{opt}**	**COT_{min}**
1	Untagged	332	140.77 ± 16.90 (***)	0.44 ± 0.06 (***)	<0.001	0.59	2.29	166.91
2	Untagged	335	103.55 ± 8.63 (***)	0.58 ± 0.04 (***)	<0.001	0.88	1.71	164.14
3	Untagged	339	132.29 ± 13.98 (***)	0.50 ± 0.06 (***)	<0.001	0.71	2.00	179.73
4	Untagged	389	114.18 ± 12.58 (***)	0.53 ± 0.05 (***)	<0.001	0.92	1.87	165.72
5	Untagged	437.8	148.93 ± 5.29 (***)	0.30 ± 0.02 (***)	<0.001	0.96	3.38	119.94
6	Untagged	437.6	148.19 ± 13.73 (***)	0.30 ± 0.05 (***)	<0.001	0.76	3.37	119.57
7	Untagged	424	88.28 ± 12.90 (***)	0.61 ± 0.07 (***)	<0.001	0.72	1.64	146.54
8	Untagged	432	149.63 ± 8.56 (***)	0.40 ± 0.03 (***)	<0.001	0.91	2.50	162.77
9	Untagged	407	97.19 ± 6.92 (***)	0.46 ± 0.03 (***)	<0.001	0.93	2.19	120.79
10	Untagged	501.2	144.75 ± 11.28 (***)	0.37 ± 0.04 (***)	<0.001	0.81	2.68	146.75
11	Untagged	589.3	118.48 ± 8.97 (***)	0.36 ± 0.04 (***)	<0.001	0.89	2.78	116.05
12	Untagged	589.3	103.92 ± 10.32 (***)	0.52 ± 0.05 (***)	<0.001	0.84	1.92	146.74
13	Untagged	502	165.54 ± 6.79 (***)	0.290.03 (***)	<0.001	0.96	3.46	130.14

14	Untagged	504	76.86 ± 14.47 (**)	0.63 ± 0.10 (**)	<0.001	0.69	1.59	131.67
15	Untagged	640.4	88.63 ± 6.83 (***)	0.49 ± 0.04 (***)	<0.001	0.94	2.04	118.14
16	Untagged	640.21	131.18 ± 8.45 (***)	0.40 ± 0.04 (***)	<0.001	0.92	2.48	143.85
17	Untagged	644.89	176.87 ± 21.30 (***)	0.34 ± 0.06 (**)	<0.001	0.88	2.93	163.91
18	Untagged	642	120.01 ± 12.79 (***)	0.49 ± 0.07 (***)	<0.001	0.81	2.06	158.50
19	Untagged	794.86	145.62 ± 9.11 (***)	0.41 ± 0.04 (***)	<0.001	0.92	2.44	161.91
20	Untagged	782.5	144.07 ± 20.81 (***)	0.39 ± 0.09 (**)	<0.001	0.49	2.56	152.77
21	Untagged	830.77	145.53 ± 7.35 (***)	0.39 ± 0.03 (***)	<0.001	0.95	2.58	153.17
22	Untagged	805	140.29 ± 7.37 (***)	0.30 ± 0.03 (***)	<0.001	0.96	3.38	112.75
23	Untagged	893.6	129.97 ± 10.09 (***)	0.43 ± 0.05 (***)	<0.001	0.89	2.33	151.39
24	Untagged	911.3	138.36 ± 7.40 (***)	0.33 ± 0.04 (***)	<0.001	0.95	3.01	124.80
25	Untagged	955	120.50 ± 6.68 (***)	0.40 ± 0.04 (***)	<0.001	0.96	2.47	132.52
1	Tagged	219	170.65 ± 12.18 (***)	0.41 ± 0.03 (***)	<0.001	0.77	2.46	188.45
2	Tagged	288.4	105.90 ± 7.64 (***)	0.47 ± 0.03 (***)	<0.001	0.91	2.12	135.69
3	Tagged	368	116.34 ± 8.24 (***)	0.42 ± 0.03 (***)	<0.001	0.89	2.37	133.51
4	Tagged	379	146.39 ± 4.35 (***)	0.33 ± 0.016 (***)	<0.001	0.98	3.03	131.47
5	Tagged	321.2	100.84 ± 16.35 (***)	0.56 ± 0.07 (***)	<0.001	0.53	1.79	153.01
6	Tagged	378	104.90 ± 9.09 (***)	0.52 ± 0.04 (***)	<0.001	0.86	1.91	149.11
7	Tagged	378	93.50 ± 8.81 (***)	0.56 ± 0.04 (***)	<0.001	0.86	1.80	141.48
8	Tagged	447	130.38 ± 9.68 (***)	0.52 ± 0.05 (***)	<0.001	0.88	1.91	185.30
9	Tagged	575	139.81 ± 16.62 (***)	0.37 ± 0.07 (**)	<0.001	0.70	2.69	141.22
10	Tagged	560	151.58 ± 18.48 (***)	0.43 ± 0.07 (***)	<0.001	0.59	2.32	177.82
11	Tagged	675	135.30 ± 10.36 (***)	0.50 ± 0.05 (***)	<0.001	0.87	1.99	185.04
12	Tagged	660.2	165.30 ± 16.53 (***)	0.37 ± 0.07 (**)	<0.001	0.73	2.70	166.37
13	Tagged	605.86	176.67 ± 12.64 (***)	0.37 ± 0.05 (***)	<0.001	0.82	2.73	175.66
14	Tagged	777.87	108.12 ± 16.79 (**)	0.55 ± 0.09 (**)	<0.001	0.64	1.80	162.86
15	Tagged	738	153.63 ± 7.34 (***)	0.42 ± 0.03 (***)	<0.001	0.94	2.40	174.20
16	Tagged	738	93.65 ± 11.66 (***)	0.62 ± 0.07 (***)	<0.001	0.81	1.61	158.16
17	Tagged	747.59	117.56 ± 11.83 (***)	0.49 ± 0.06 (**)	<0.001	0.85	2.05	155.62
18	Tagged	747.59	198.11 ± 12.07 (***)	0.26 ± 0.03 (**)	<0.001	0.98	3.87	139.18
19	Tagged	851.7	142.10 ± 9.04 (***)	0.36 ± 0.05 (**)	<0.001	0.94	2.77	139.40
20	Tagged	885	107.17 ± 9.86 (***)	0.45 ± 0.06 (***)	<0.001	0.90	2.23	130.71
21	Tagged	885	94.33 ± 6.61 (***)	0.45 ± 0.04 (***)	<0.001	0.96	2.21	115.87
22	Tagged	837	109.50 ± 15.65 (***)	0.47 ± 0.07 (***)	<0.001	0.69	2.12	140.22
23	Tagged	803	121.16 ± 6.00 (***)	0.37 ± 0.03 (***)	<0.001	0.97	2.73	120.54
24	Tagged	803	87.32 ± 4.96 (***)	0.50 ± 0.03 (***)	<0.001	0.97	2.01	117.97
25	Tagged	935	116.14 ± 14.40 (**)	0.58 ± 0.08 (**)	<0.001	0.78	1.72	183.66
26	Tagged	971	134.85 ± 5.76 (***)	0.34 ± 0.03 (***)	<0.001	0.97	2.97	123.44
27	Tagged	902	146.59 ± 4.90 (***)	0.34 ± 0.02 (***)	<0.001	0.98	2.91	137.08

Table S5. Outputs of exponential model for fitting the swimming activity (arbitrary unit, AU) recorded by tag as a function of swimming speed (m/s) during Ucrit trial for each Gilt-head Sea bream (Sparus aurata; n=23 tagged fish). P value and R^2 were shown for each model. Estimate, standard error and level of significance are presented for each parameter of the model (α and β). For the level of significance, not significant (NS) is indicated for p>0.1, # for 0.05 <p<0.1, * for p<0.05, ** for p<0.01 and *** for p<0.001.

		Estimate ± std. error (significance)			
**Fish ID**	**Mass**	**α**	**β**	**p value**	**R^2**
1	219	17.93 ± 3.20 (***)	1.84 ± 0.18 (***)	<0.001	0.98
2	288.4	5.59 ± 1.25 (***)	3.27 ± 0.23 (***)	<0.001	0.99
3	368	12.65 ± 1.75 (***)	2.39 ± 0.13 (***)	<0.001	0.98
4	379	4.37 ± 0.80 (***)	3.41 ± 0.17 (***)	<0.001	0.99
5	321.2	9.16 ± 1.67 (***)	2.47 ± 0.18 (***)	<0.001	0.99
6	378	7.27 ± 1.20 (***)	3.13 ± 0.17 (***)	<0.001	0.99
7	378	9.28 ± 1.66 (***)	2.82 ± 0.17 (***)	<0.001	0.98
8	447	14.67 ± 4.56 (*)	2.79 ± 0.37 (***)	<0.001	0.93
9	575	8.88 ± 1.31 (***)	2.88 ± 0.15 (***)	<0.001	0.99
10	560	19.52 ± 4.91 (**)	2.45 ± 0.26 (***)	<0.001	0.91
11	675	11.02 ± 2.41 (**)	3.17 ± 0.25 (***)	<0.001	0.97
12	660.2	12.84 ± 2.58 (**)	2.86 ± 0.23 (***)	<0.001	0.98
14	777.87	6.00 ± 1.41 (**)	3.86 ± 0.30 (***)	<0.001	0.99
15	738	7.72 ± 1.34 (**)	3.45 ± 0.19 (***)	<0.001	0.99
16	738	13.99 ± 2.10 (**)	2.43 ± 0.20 (***)	<0.001	0.99
17	747.59	9.23 ± 3.08 (*)	3.25 ± 0.36 (***)	<0.001	0.94
18	747.59	3.63 ± 0.84 (**)	4.23 ± 0.25 (***)	<0.001	0.99
19	851.7	9.14 ± 2.25 (**)	3.16 ± 0.32 (***)	<0.001	0.98

22	837	11.55 ± 2.64 (**)	2.95 ± 0.26 (***)	<0.001	0.97
23	803	15.19 ± 3.04 (**)	2.45 ± .23 (***)	<0.001	0.98
24	803	15.04 ± 2.30 (***)	2.36 ± 0.18 (***)	<0.001	0.99
25	935	24.53 ± 2.01 (***)	1.98 ± 0.11 (***)	<0.001	1.00
26	971	14.14 ± 1.90 (***)	2.52 ± 0.13 (***)	<0.001	0.98
